# Advances in application of hypoxia-preconditioned mesenchymal stem cell-derived exosomes

**DOI:** 10.3389/fcell.2024.1446050

**Published:** 2024-08-21

**Authors:** Haitao Zhuo, Yunfei Chen, Guifang Zhao

**Affiliations:** ^1^ The Affiliated Qingyuan Hospital (Qingyuan People’s Hospital), Guangzhou Medical University, Qingyuan, China; ^2^ Department of Nuroscience, Mayo Clinic, Jacksonville, FL, United States; ^3^ Department of Pathology, Jilin Medical University, Jilin, China

**Keywords:** hypoxic preconditioning, mesenchymal stem cells, exosomes, microRNA, molecular mechanism

## Abstract

Mesenchymal stem cells (MSCs) primarily secrete physiologically functional exosomes via paracrine effects that act on various adjacent and distant cells, thus exerting their therapeutic effects. In recent years, hypoxic preconditioning, as a novel MSC culture mode, has emerged as a research hotspot. Many previous studies have shown the role and underlying regulatory mechanisms of hypoxic preconditioning in various diseases, which has provided sufficient reference materials for the MSC research field. Therefore, this review summarizes the progress in application of hypoxia-preconditioned MSC-derived exosomes that substantially increases and improves the biological activity of specific molecules, such as microRNA.

## 1 Introduction

Mesenchymal stem cells (MSCs) are pluripotent cells with self-renewal and differentiation properties that are widely distributed in many parts of the human body (Bianco and Gehron Robey, 2000). These include bone marrow, adipose tissue, dental pulp, umbilical cord and placenta, among others (Bianco and Gehron Robey, 2000). The efficacy of stem cell therapy has been well documented in numerous preclinical studies (Uccelli et al., 2008). However, the therapy’s limitations, including low implantation rates (Fouillard et al., 2007) and implantation side effects (Fennema et al., 2018; Jeong et al., 2011; Kusuma et al., 2015; Wang et al., 2015) have prompted researchers to seek improvements.

Exosomes are currently one of the most popular areas of research in the field of stem cells. Exosomes are biological nanovesicles with a diameter of 30–150 nm, secreted by different types of cells, containing messenger molecules, including coding and non-coding RNAs, which can regulate the functions of proximal and distal cells (Lai et al., 2015). The term “proximal” refers to functions inside the cell, such as metabolism and signalling, while “distal” refers to functions outside the cell, such as intercellular communication and tissue repair. It can be posited that the role of mesenchymal stem cell-derived exosomes (MSC-Exos) is to regulate and influence intra- and extracellular functions in order to maintain tissue homeostasis and promote tissue repair. Furthermore, it can be hypothesised that MSC-Exos possess the ability to interact with a wide range of cell types in neighbouring and remote regions, thereby triggering appropriate cellular responses. Although exosomes are characterised by low toxicity, high stability, low immunogenicity and efficient transport of donor cells (Zhu J. et al., 2018), the therapeutic use of exosomes is limited by the limited number of cells from which they are derived and the different components they secrete. Consequently, the current scientific challenge in the field of MSC-Exos applications is to identify effective methods for optimising the therapeutic effects of MSC-Exos. Recent studies have indicated that hypoxic pretreatment of MSCs represents an effective strategy for modulating their therapeutic potential, resulting in the release of exosomes with enhanced biological functions through paracrine effects. The low oxygen concentration required for MSC culture was preset according to the tissue source, and the exosomes obtained from the hypoxic pretreated MSCs were extracted within a specified time period, designated as hypoxic MSC-Exos (HypMSC-Exos). This paper reviews the progress of the application of HypMSC-Exos.

## 2 Overview of HypMSC-Exos

Exosomes are lipid bilayer vesicles with a diameter of 30–150 nm, which carry a variety of substances, including lipids, metabolites, proteins, microRNAs (miRNAs), mRNAs, long noncoding RNAs (lncRNAs) and DNA (Pegtel and Gould, 2019). Previous studies have demonstrated that HypMSC-Exos enhance mRNA expression and secretion of several crucial cytokines, including vascular endothelial growth factor (VEGF) and basic fibroblast growth factor. Notably, this hypoxic preconditioning did not result in any adverse effects on the release of other cytokines (Kinnaird et al., 2004). It was subsequently demonstrated that hypoxic pre-acclimatisation augmented the protective factors of MSC-Exos against future hypoxia-injured tissues, including hypoxia-inducible factor-1α (HIF-1α), angiogenic factors (VEGF, angiopoietin 1 and erythropoietin), survival proteins (P65, P50, and P105) and anti-apoptotic proteins (Bcl-xL and Bcl-2) expression (Hu et al., 2008). A considerable number of studies have demonstrated that hypoxic preconditioning of mesenchymal stem cells (MSCs) modulates the expression of specific molecules and enhances the therapeutic effect of MSC-derived exosomes (MSC-Exos). Among these, microRNAs (miRNAs) have received particular attention due to their pivotal role in regulating gene expression. One study demonstrated that 215 miRNAs were upregulated and 369 miRNAs were downregulated in HypMSC-Exos in comparison to normoxic exosomes (Casado-Díaz et al., 2020; Zhang et al., 2017). Among the observed effects, HypMSC-Exos demonstrated a notable increase in the expression of miRNAs with specific functions, indicating a potential joint role for miRNAs in the observed processes (Liang et al., 2022). Consequently, these studies indicate that miRNAs in exosomes play a pivotal role in intracellular signalling, transport and cellular therapy *in vitro* (Ong et al., 2014; Gray et al., 2015).

## 3 Conditions of hypoxic pre-treatment of MSCs

**FIGURE 1:**
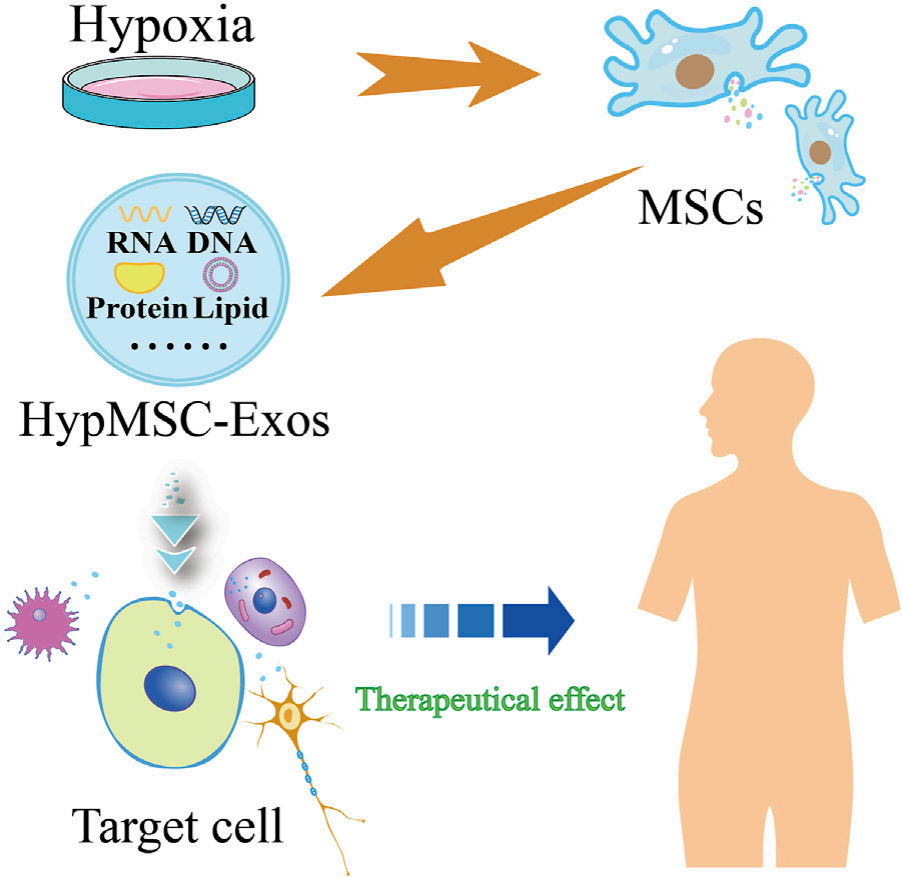
Flow chart of hypoxic pretreatment of mesenchymal stem cells (MSCs) to produce hypoxic MSC-derived exosomes (HypMSC-Exos).

A substantial body of evidence indicates that the characteristics of mesenchymal stem cells (MSCs) are influenced by alterations in environmental conditions. It has been demonstrated that changes in atmospheric oxygen concentrations may result in DNA damage to cellular components and may consequently lead to genetic instability (Estrada et al., 2012), which may in turn result in differences in the therapeutic efficacy of MSCs. *In vitro* culture of MSCs typically occurs under normoxic conditions (21% O₂). However, it is known that MSCs are in a hypoxic ecological niche *in vivo*, and that the majority of MSCs exist in environments with 2%–8% (or even lower) oxygen content *in vivo* (Xue et al., 2018). Consequently, culturing MSCs in a low-oxygen environment would be more closely aligned with their physiological environment. There is accumulating evidence that maintaining MSCs under low partial pressure of oxygen may assist in preserving their characteristics and enhancing their therapeutic potential. For instance, reduced oxygen supply is a common occurrence in damaged tissues. Once MSCs migrate to the hypoxic zone, their paracrine effect produces a substantial quantity of therapeutic paracrine factors to repair damaged tissues (Han et al., 2019). Furthermore, it has been demonstrated that hypoxic preconditioning or the activation of HIF-1α expression *in vitro* reduces the apoptosis of bone marrow mesenchymal stem cells (BMMSCs) cultured *in vitro*, thus playing an important role in the survival of BMMSCs after transplantation (Luo et al., 2019). Consequently, hypoxic preconditioning can be employed as a therapeutic strategy to enhance the growth environment of MSCs and stimulate the secretion of exosomes with physiological functions (Figure 1).

## 4 Role of HypMSC-Exos

### 4.1 Promoting angiogenesis and repair

A number of studies have demonstrated the importance of HypMSC-Exos in the field of angiogenesis and repair. The mechanism by which HypMSC-Exos exerts its effects may be to promote angiogenesis and repair by altering the miRNA and proteomic profiles and modulating multiple signalling pathways. An early study demonstrated that hypoxic preconditioning could enhance the promotion of neovascularisation in transplanted adipose tissue by hypoxic adipose mesenchymal stem cell-derived exosomes (HypADSC-Exos) (Han et al., 2018). The results of this experiment indicated that the composition of HypMSC-Exos was altered by the hypoxic environment, which enhanced the angiogenic potential of MSC-Exos. This was evidenced by the alteration of their proteomic profile (Li et al., 2022).

A significant number of studies have demonstrated that the HIF-1α or VEGF signalling pathway is a downstream target of HypMSC-Exos, which enhances angiogenesis. The key signalling molecules and upstream signalling pathways are currently being investigated. For instance, the up-regulation of high mobility group box 1 (HMGB1) in HypMSC-Exos mediates the expression of the c-Jun N-terminal kinase (JNK) pathway activation, which induces the expression of HIF-1α/VEGF, thereby promoting angiogenesis (Gao et al., 2021; Chen and Zhang, 2021). Furthermore, extracellular vesicles derived from hypoxic mucosal MSCs have been shown to upregulate miR-612 by inhibiting tumour protein 53 (Tp53), thereby inducing the expression of the HIF-1α/VEGF pathway, which promotes the generation of microvascular endothelial cells in the human brain (Ge et al., 2021). Furthermore, studies have demonstrated that HypADSC-Exo may facilitate angiogenesis by activating the protein kinase A signalling pathway and promoting the expression of the VEGF pathway (Xue et al., 2018). HIF-1α is a crucial transcription factor for MSCs in hypoxic environments. Studies have demonstrated that hypoxic conditions may markedly enhance the pro-angiogenic effects of MSC-Exos by mediating the activation of miR-210 and neutral sphingomyelinase 2 through the action of HIF-1α (Zhu J. et al., 2018). Nevertheless, a few articles have also reported the existence of other signalling pathways regulated by HypMSC-Exos. It has been demonstrated that the expression of let-7f-5p and miR-210-3p is elevated in exosomes derived from hypoxia-pretreated human deciduous tooth stem cells. This upregulation has been shown to promote angiogenesis via two distinct pathways: the let-7f-5p/AGO1 (argonaute RISC component 1)/VEGF pathway and the miR-210-3p/ephrinA3 pathway (Liu P. et al., 2022). This suggests that they may become a novel signalling pathway for pro-angiogenic therapy with HypMSC-Exos. Hypoxic preconditioning represents an effective method for enriching pro-angiogenic factors in MSC-Exos. HypMSC-Exos have been demonstrated to promote angiogenesis by upregulating the expression of LOXL2 (lysyl oxidase-like 2), CXCR4 (C-X-C chemokine receptor 4) and SDF-1 (stromal cell-derived factor-1) (Li et al., 2022), although the specific molecular mechanisms remain to be elucidated. The aforementioned findings indicate that HIF-1α may play a pivotal role in the regulation of the signalling pathway by HypMSC-Exos. However, the precise mechanism by which hypoxia influences the release of MSC-Exos remains elusive. In conclusion, HypMSC-Exos may enhance the effect of angiogenesis. Therefore, future research should focus on studying the upstream and downstream signalling pathway regulation mechanisms of molecules such as miRNAs and proteins.

### 4.2 Promoting repair of injured myocardium

The burden of heart failure arising after myocardial infarction remains a significant problem in clinical practice. A growing number of studies have demonstrated that HypMSC-Exos significantly improves ischemic cardiomyocyte apoptosis, promotes their remodelling, and protects cardiac function. A study demonstrated that hypoxic umbilical cord mesenchymal stem cell-derived exosomes (HypUCMSC-Exos) significantly promoted the repair of injured myocardium after myocardial infarction in SD rats (Zhang P. et al., 2019). The changes of miRNAs and lncRNAs in HypUCMSC-Exos are important key regulatory signals in the study of myocardial injury repair mechanisms. Hypoxic BMMSC-derived exosomes (HypBMMSC-Exos) have been demonstrated to inhibit apoptosis of rat cardiomyocytes following acute infarction by upregulating miR-24 (Zhang C. S. et al., 2019). Furthermore, miR-125b-5p in HypMSCExos inhibits cardiomyocyte apoptosis by suppressing the expression of P53 and BAK1 (BCL2 antagonist/killer 1), thereby promoting ischaemic heart repair (Zhu L. P. et al., 2018). Additionally, miR-98-5p within HypBMMSC-Exos mediates the inhibition of myocardial ischaemia-reperfusion injury by targeting TLR4 through the activation of the PI3K/Akt signalling pathway (Zhang L. et al., 2021). It has been demonstrated that HypMSC-Exos upregulate the expression of lncRNA-UCA1, which in turn activates XIAP (X-linked inhibitor of apoptosis protein) to exert cardioprotective effects through the inhibition of miR-873-5p (Sun et al., 2020), suggesting that exosomal lncRNA-UCA1 may be a promising new biomarker for cardioprotection. In a further study, Liu et al. (2019) demonstrated that HypUCMSC-Exo could regulate autophagy through the PI3K/Akt/mTOR pathway, thereby inhibiting apoptosis in myocardial H9C2 cells induced by hypoxia and serum deprivation. A limited number of articles have also reported the study of HypMSC-Exos against adriamycin-induced myocardial injury. HypMSC-Exos has been demonstrated to counteract adriamycin-induced myocardial injury through two distinct pathways: the lncRNA-MALAT1/miR-92a-3p/ATG4a (autophagy-related 4a cysteine peptidase) axis and the Trx1 (thioredoxin 1)/mTORC1 signalling pathway. These pathways have been shown to counteract adriamycin-induced cardiomyocyte damage and senescence, thereby facilitating cardiac recovery (Xia et al., 2020; Yu et al., 2022). This further demonstrates the therapeutic potential of HypMSC-Exos for acute cardiotoxicity. In summary, HypMSC-Exos represents a promising therapeutic approach for the treatment of tissue ischaemia and cardiotoxic injury. However, the specific targeting of exosomes to the ischaemic heart remains a challenge.

### 4.3 Promoting bone repair and regeneration

HypMSC-Exos has been demonstrated to have significant therapeutic effects on a number of conditions affecting bone, including hormonal osteonecrosis of the femoral head (Yuan et al., 2021), bioactive fibrous ring repair (DiStefano et al., 2022), tendon-bone tunnel healing (Zhang T. et al., 2022), and other bony injuries. However, the exact molecular mechanism of the therapeutic effect remains to be investigated. Liu et al. (2020a) demonstrated that the hypoxic environment may significantly enhance fracture healing through the action of HIF-1α, which mediates the up-regulation of miR-126. This significantly improves fracture healing. In the field of cartilage regeneration engineering, the incorporation of hydrogel-embedded HypMSC-Exos has been demonstrated to promote cartilage regeneration through the up-regulation of miR-205-5p expression and the activation of the PTEN/AKT pathway (Shen et al., 2022), thereby suggesting that the composite of HypMSC-Exos and silk fibroin hydrogel has a broad application prospect in cartilage regeneration. In the meantime, bioinformatics analysis indicated that hypoxia pretreatment of extracellular vesicles derived from mesenchymal stem cells (MSCs) may stimulate the proliferation and migration of chondrocytes and inhibit the apoptosis of chondrocytes via the miR-18-3p/JAK/STAT or miR-181c-5p/MAPK signalling pathways, thus promoting cartilage repair (Zhang B. et al., 2022). Recent studies have demonstrated that exosomes secreted by HypADSC-Exos and tendon cells protect the tendon matrix from damage by releasing regenerative mediators into the extracellular matrix, thereby triggering an autocrine/paracrine response (Thankam et al., 2020). This study also demonstrated that certain mRNA and protein levels in these two exosomes are in a state of dynamic equilibrium, and that they are involved in a multitude of signalling pathways that pertain to the extracellular matrix. Furthermore, the study demonstrated that certain mRNAs and proteins in the two exocrine species exhibited dynamic equilibrium, and were implicated in a multitude of extracellular matrix-related signaling pathways. In conclusion, hypoxic preconditioning represents an effective and promising method to optimise the therapeutic effects of MSC-Exos on bone repair and regeneration.

### 4.4 Promoting nerve repair and regeneration

Nerve injuries can result in severe motor and sensory dysfunction, with high rates of disability and mortality. An initial study observed that HypMSC-Exos facilitated cerebral remodelling and neurological recovery in mice following focal cerebral ischaemia (Gregorius et al., 2021). Nevertheless, the optimal therapeutic strategy for neurological injury is the polarization transition of microglia and the suppression of deleterious, excessive neuroinflammation. It has now been demonstrated that HypMSC-Exos plays a more efficacious therapeutic role in the polarisation transition of microglia and represents a promising therapeutic target in the field of neurological injury. For instance, (1) HypBMMSC-Exos enriched with miR-216a-5p facilitates the transition of microglia from the M1 to the M2 phenotype by inhibiting the TLR4/NF-κB pathway and activating the PI3K/Akt signalling pathway (Liu et al., 2020b). (2) The up-regulation of lncRNA-Gm37494 expression in HypADSC-Exos has been demonstrated to promote microglia M1/M2 polarisation by inhibiting miR-130b-3p and promoting PPARγ expression (Shao et al., 2020). (3) HypADSC-Exos reduces miR-7703p expression, thereby altering microglia M1/M2 polarisation and improving cognition in Alzheimer’s disease mice. This is achieved by delivering circ-Epc1-mediated TREM2 expression (Liu H. et al., 2022). (4) HypADSC-Exos inhibited miR-124-3p by delivering circ-Rps5 to promote SIRT7 expression, thereby attenuating acute ischemic stroke-induced brain injury and promoting microglia M1/M2 polarisation (Yang H. et al., 2022). In addition, HypBMMSC-Exos demonstrated robust protective effects against apoptosis in neuronal injury. For instance, HypBMMSC-Exos has been demonstrated to repair oxygen and sugar deprivation-induced neuronal injury by inhibiting NLRP3 inflammatory vesicle-mediated caspase death (Kang et al., 2021). (2) MiR-499-5p within HypADSC-Exos plays a pivotal role in regulating neuronal apoptosis by negatively regulating the JNK3/MAPK10/c-Jun signalling pathway, thereby reducing neuronal apoptosis in rat neuronal cells (Liang et al., 2022). (3) circOXNAD1 within HypUCMSC-Exos activates FOXO3a expression, increases neuronal viability, and inhibits cell death and inflammation by parceling out miR-29a-3p (Wang et al., 2023). In materials engineering, the combined use of HypMSCExos and 3D-CS scaffolds has been demonstrated to promote nerve regeneration after traumatic brain injury, while inhibiting chronic neuroinflammation and neural apoptosis (Liu X. et al., 2022). This indicates that the combination of HypMSC-Exos therapy with carrier materials represents a novel approach to the treatment of traumatic brain injury.

### 4.5 Promoting the repair of skin injuries

Poor healing of skin wounds can be life-threatening and is a significant public health concern globally. In a previous study, Qiu et al. (2020) demonstrated that MSC-Exos exhibited a more pronounced therapeutic effect on skin wound healing, suggesting a promising avenue for cell-free treatment of skin injury. Among the various types of skin injury, diabetic wounds are particularly challenging to heal due to persistent inflammation and delayed healing caused by hypoxia. HypADSC-Exos has been shown to promote fibroblast proliferation and migration through the activation of the PI3K/Akt pathway, which can accelerate the rate of diabetic wound healing and improve the quality of wound healing. Additionally, HypADSC-Exos has been demonstrated to inhibit inflammatory responses (Wang et al., 2021). Zhang X. F. et al. (2021) demonstrated that miR-125b upregulation within HypUCMSC-Exos enhances endothelial cell survival and migration during wound healing by targeting and inhibiting TP53INP1 (tumor protein p53 inducible nuclear protein 1). Furthermore, HypADSC-Exos facilitates wound healing in diabetic mice by delivering circ-Snhg11 and activating the miR-144-3p/HIF-1α pathway (Shi et al., 2022). In conclusion, HypMSC-Exos represents a promising area for further investigation in the context of diabetic wound healing. Consequently, future research should prioritise the exploration of additional signalling pathway mechanisms associated with HypMSC-Exos.

### 4.6 Promoting the regulation of inflammation

A number of studies have demonstrated that HypMSC-Exos has been shown to have more positive therapeutic effects in modulating inflammation compared to normoxic exosomes in basic research. Yang T. et al. (2022) demonstrated that HypBMMSC-Exos up-regulated the expression of HIF-1α and activated the antioxidant signalling of superoxide dismutase to attenuate the ototoxicity of cisplatin and mitigate excessive oxidative stress. This study suggests that HypMSC-Exos may have a potential preventive effect on severe oxidative stress. The up-regulation of miR-182-5p within HypBMSC-Exo has been demonstrated to promote macrophage polarisation through modulation of the FOXO1/TLR4 signalling pathway. This evidence supports the therapeutic potential of HypMSC-Exos in liver regeneration, as outlined in reference (Xu et al., 2022). Recent studies have shown that the up-regulation of miR-216a-5p in HypADSC-Exos has the dual effect of promoting macrophage M2-type polarisation through the regulation of the HMGB1/TLR4/NF-κB axis to attenuate excessive inflammation in colitis (Qian et al., 2023) and of regulating the accumulation of reactive oxygen species in intestinal epithelial cells, DNA damage, and immune homeostasis by HIF-1α, thereby attenuating mucosal damage in ulcerative colitis (Zhu et al., 2022). Mucosal damage is a well-documented consequence of ulcerative colitis. This indicates that HypMSC-Exos represents a promising avenue for optimising colitis treatment. Furthermore, Cao et al. (2022) observed and confirmed that HypADSC-Exos, which highly express mmu_circ_0001295, improved the outcome and inhibited sepsis-induced kidney injury in septic mice. This study demonstrates the feasibility of MSC-Exos as a novel target for the treatment of sepsis-induced kidney injury.

## 5 Summary and outlook

**FIGURE 2:**
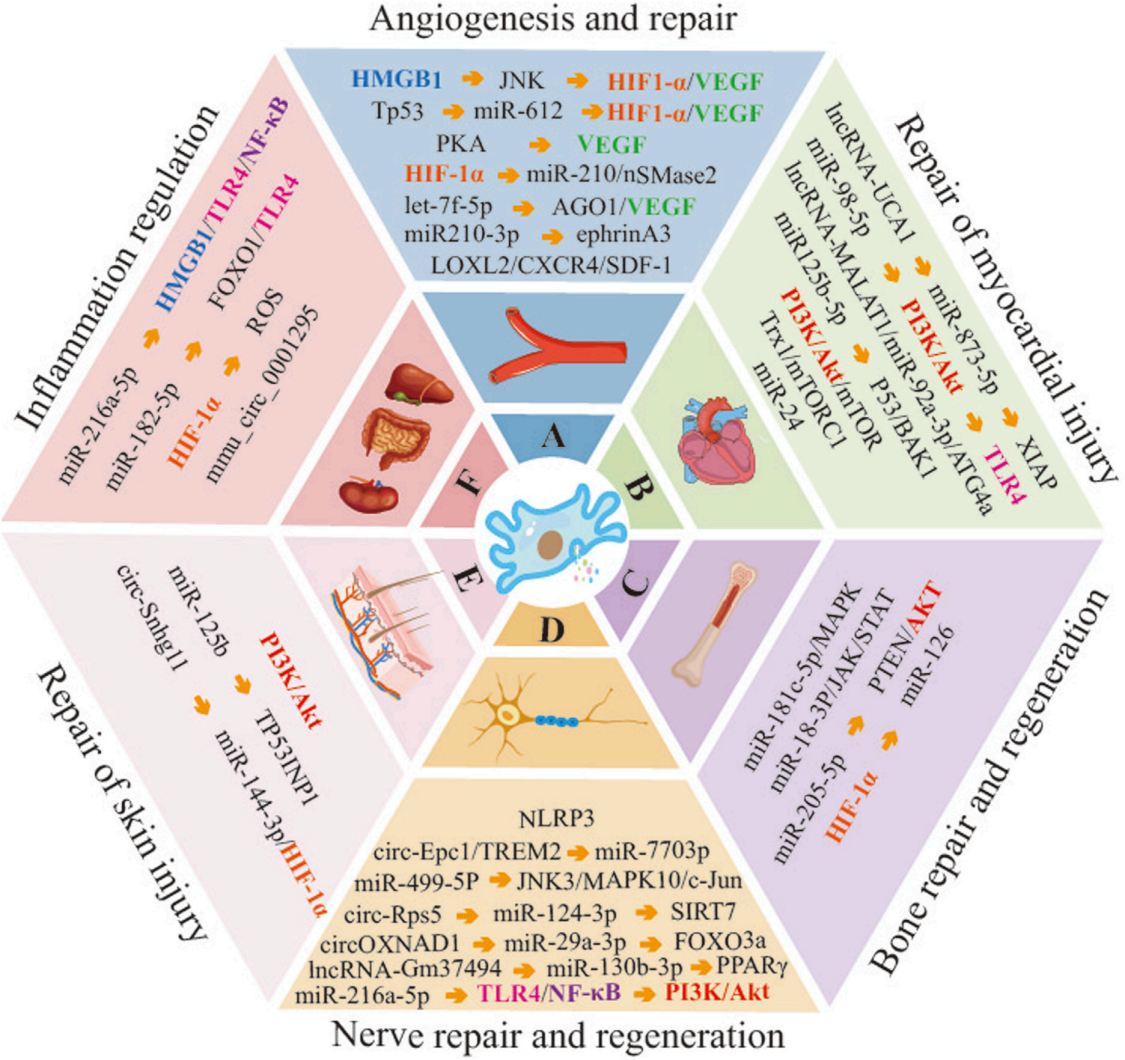
Molecular mechanisms of HypMSC-Exos regulation of related disease progression. (Abbreviations: HMGB1, high mobility group box 1; JNK, c-Jun N-terminal kinase; HIF-1α, hypoxia-inducible factor-1α; VEGF, vascular endothelial growth factor; Tp53, tumor protein 53; PKA, protein kinase A; nSMase2, neutral sphingomyelinase 2; AGO1, argonaute RISC component 1; LOXL2, lysyl oxidase-like 2; CXCR4, C-X-C chemokine receptor 4; SDF-1, stromal cell-derived factor-1; PI3K, phosphatidylinositol 3-kinase; TLR4, Toll-like receptor 4; ATG4a, autophagy related 4a cysteine peptidase; BAK1, BCL2 antagonist/killer 1; Trx1, thioredoxin 1; mTORC1, mammalian target of rapamycin complex 1; MARK, mitogen-activated protein kinase; JAK, janus kinase; STAT, signal transducer and activator of transcription; PTEN, phosphatase and tensin homolog; NLRP3, NOD-like receptor family, pyrin domain containing 3; Epc1, enhancer of polycomb homolog 1; TREM2, triggering receptor expressed on myeloid cells 2; SIRT7, sirtuin 7; FOXO3a, forkhead box O3; PPARγ, peroxisome proliferator-activated receptor γ; TP53INP1, tumor protein p53 inducible nuclear protein 1).

This paper presents a comprehensive overview of the progress made in the application of hypoxic pretreatment on the therapeutic role of MSC-Exo. A significant body of evidence from numerous studies indicates that HypMSC-Exos exhibit remarkable therapeutic potential in promoting angiogenesis and repair, myocardial repair, and other areas (Figure 2). This suggests that exploring the optimal conditions of hypoxic pretreatment of MSCs is an effective strategy for enhancing the biological functions of MSC-Exos, which in turn contributes to the effectiveness of exosome therapy. Nevertheless, the utilisation of hypoxic preconditioning in MSC-Exos is still in its nascent stages and is subject to numerous unresolved issues. For instance, it is necessary to determine the optimal concentration of hypoxia for MSCs of different tissue origins. (2) What are the differences in exosomes secreted by MSCs from different tissue sources under the same hypoxic preconditioning conditions? Does the administration of MSCs from different tissue sources result in different outcomes? (3) Currently, the majority of experiments investigating HypMSC-Exos are conducted at the cellular level. Therefore, further research is required to investigate the administration and dosage of HypMSC-Exos in animal experiments. (4) The current research on HypMSC-Exos is focused on the internal miRNAs, while the biological potential of the secreted proteins, lipids and other non-coding RNAs still requires further elucidation. (5) Following systemic administration, it remains challenging to elucidate the specific targeting mechanism of HypMSC-Exos to target organs, tissues and cells. Furthermore, the potential of HypMSC-Exos as a nanocarrier or active factor in tissue engineering and regenerative medicine requires further investigation. In conclusion, a comprehensive examination of the aforementioned pertinent issues will facilitate the enhanced efficacy of HypMSC-Exos in clinical exosome-based therapies for various diseases.
