# Corrigendum: Advances in application of hypoxia-preconditioned mesenchymal stem cell-derived exosomes

**DOI:** 10.3389/fcell.2025.1583347

**Published:** 2025-03-13

**Authors:** Haitao Zhuo, Yunfei Chen, Guifang Zhao

**Affiliations:** ^1^ The Affiliated Qingyuan Hospital (Qingyuan People’s Hospital), Guangzhou Medical University, Qingyuan, China; ^2^ Department of Nuroscience, Mayo Clinic, Jacksonville, FL, United States; ^3^ Department of Pathology, Jilin Medical University, Jilin, China

**Keywords:** hypoxic preconditioning, mesenchymal stem cells, exosomes, microRNA, molecular mechanism

In the published article, there was an error regarding the **Affiliation(s)** for Guifang Zhao. Instead of “^2,3*^”, it should be “^3*^”.

The authors apologize for this error and state that this does not change the scientific conclusions of the article in any way. The original article has been updated.

